# Mast cells promote melanoma colonization of lungs

**DOI:** 10.18632/oncotarget.11837

**Published:** 2016-09-02

**Authors:** Helena Öhrvik, Mirjana Grujic, Ida Waern, Ann-Marie Gustafson, Nancy Ernst, Axel Roers, Karin Hartmann, Gunnar Pejler

**Affiliations:** ^1^ Department of Medical Biochemistry and Microbiology, Uppsala University, Uppsala, Sweden; ^2^ Department of Anatomy, Physiology and Biochemistry, Swedish University of Agricultural Sciences, Uppsala, Sweden; ^3^ Institute for Immunology, University of Technology Dresden, Dresden, Germany; ^4^ Department of Dermatology, University of Luebeck, Luebeck, Germany

**Keywords:** mast cells, Mcpt5, melanoma, inflammation, EMT

## Abstract

Mast cells have been implicated in malignant processes, mainly through clinical correlative studies and by experiments performed using animals lacking mast cells due to defective c-kit signaling. However, mast cell-deficient mouse models based on c-kit defects have recently been questioned for their relevance. Here we addressed the effect of mast cells in a tumor setting by using transgenic Mcpt5-Cre^+^ R-DTA^+^ mice, in which the deficiency of mast cells is independent of c-kit defects. Melanoma cells (B16.F10) were administered either subcutaneously or intravenously into Mcpt5-Cre^+^ R-DTA^+^ mice or Mcpt5-Cre^−^ R-DTA^+^ littermate controls, followed by the assessment of formed tumors. In the subcutaneous model, mast cells were abundant in the tumor stroma of control mice but were absent in Mcpt5-Cre^+^ R-DTA^+^ mice. However, the absence of mast cells did not affect tumor size. In contrast, after intravenous administration of B16.F10 cells, melanoma colonization of the lungs was markedly reduced in Mcpt5-Cre^+^ R-DTA^+^ vs. Mcpt5-Cre^−^ R-DTA^+^ animals. Decreased melanoma colonization of the lungs in Mcpt5-Cre^+^ R-DTA^+^ animals was accompanied by increased inflammatory cell recruitment into the bronchoalveolar lavage fluid, suggesting that mast cells suppress inflammation in this setting. Further, qPCR analysis revealed significant alterations in the expression of Twist and E-cadherin in lungs of Mcpt5-Cre^+^ R-DTA^+^ vs. control Mcpt5-Cre^−^ R-DTA^+^ animals, suggesting an impact of mast cells on epithelial-mesenchymal transition. In conclusion, this study reveals that mast cells promote melanoma colonization of the lung.

## INTRODUCTION

Mast cells are well known for their devastating impact on allergic reactions, the most serious manifestation being anaphylactic shock [1, 2]. However, mast cells have additionally been implicated as detrimental players in numerous disorders of non-allergic nature, for example atherosclerosis [3], arthritis [4], skin blistering [5], nephritis [6] and also in cancer (reviewed in [7-10]).

A role for mast cells in cancer has been documented in a vast number of clinical studies, where mast cell presence has been observed in a large number of different types of malignancies [7-11]. Typically, mast cells show a strong accumulation in the tumor stroma under such conditions, but mast cell presence within the tumor mass has also been documented. To address the function of mast cells in malignancies, the correlation between mast cell presence and prognosis has been studied extensively. In such correlative studies, mast cell presence has in many cases been correlated with bad prognosis, suggesting that they are detrimental. Examples of this scenario include squamous carcinomas [12, 13], nodular sclerotic-type Hodgkin's lymphoma [14], Waldenströms macroglobulinemia [15] and prostate cancer [16]. However, mast cell presence has in other cases been correlated with good prognosis, i.e. indicating that mast cells can be protective. Examples of the latter include breast cancer [17, 18], non-small-cell lung carcinoma [19], diffuse large B-cell lymphoma [20] and ovarian cancer [21]. In melanoma, partly conflicting clinical findings have been published (reviewed in [10]), with some studies indicating a detrimental impact of mast cells [22-24] whereas others have reported a positive correlation between mast cell presence and good prognosis [25].

To experimentally address the role of mast cells in malignancies, investigators have assessed various mast cell-deficient mouse models, mainly such in which mast cell-deficiency is a result of defective c-kit signaling [26-31]. However, c-kit is not only expressed in the mast cell niche and, consequently, the c-kit-defective animals can suffer from several additional abnormalities including anemia, alterations in neutrophil populations as well as deficiency of melanocytes, interstitial cells of Cajal and basophils [32]. In order to obtain mouse models that enable a more accurate assessment of mast cell functions, several laboratories have therefore generated mice in which mast cell-deficiency is independent of defects in c-kit signaling. These include strategies utilizing the Cpa3 promoter, giving rise to mice that lack mast cells but also basophils [33, 34]. In another strategy, mast cell-deficiency is driven by expression of Cre recombinase under the control of the Mcpt5 promoter [35, 36].

To probe the role of mast cells in melanoma, we here implemented the Mcpt5-Cre approach. Our findings indicate that mast cells promote melanoma colonization of lungs, thereby introducing the possibility that mast cells have a role in melanoma metastasis.

## RESULTS

### Mast cells do not influence tumor size in a subcutaneous model

In this study we used mice in which Cre recombinase expression is driven by the promoter for Mcpt5 (Mcpt5-Cre^+^ mice), Mcpt5 (also denoted mouse mast cell protease 5 (mMCP5)) being a chymase that is restricted to mast cells of the connective tissue subtype (CTMCs; [37]). These mice are crossed to the R-DTA^+^ line, harboring the diphteria toxin gene with a loxP-flanked stop cassette. Deletion of this stop cassette by Mcpt5-driven Cre recombinase expression will activate diphteria toxin expression, leading to cellular suicide [35].

**Figure 1:**
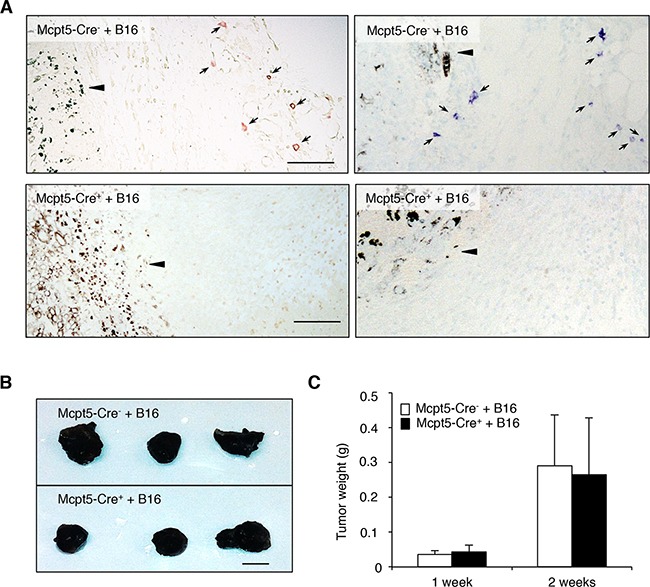
Mast cells do not influence the growth of subcutaneous melanoma tumors Melanoma cells (B16.F10) were injected subcutaneously into the flanks of either Mcpt5-Cre^−^ or Mcpt5-Cre^+^ mice. After 1 or 2 weeks, tumors were excised. **A.** Staining of subcutaneous tissue of Mcpt5-Cre^−^ or Mcpt5-Cre^+^ mice with either chloroacetate esterase (left panels) or toluidine blue (right panels). Note the presence of chloroacetate esterase- and toluidine blue-positive mast cells (marked with arrows) in tissue from Mcpt5-Cre^−^ animals (upper panels) and the absence of mast cells in tissue from Mcpt5-Cre^+^ mice (lower panels). Note also that mast cells are abundant at a distance of ∼300 μm from the tumor border (arrowhead), but not detectable within the tumor mass. Original magnification = 200 x; Bar = 100 μm. **B.** Representative images of tumors excised from Mcpt5-Cre^−^ and Mcpt5-Cre^+^ mice 2 weeks after injection of 0.5 − 10^6^ B16.F10 cells. Bar = 5 mm **C.** Tumor end weights 1 or 2 weeks after injection of 0.5 − 10^6^ B16.F10 cells. Results are shown as mean ± SD; n = 4-7.

To study the role of mast cells in melanoma we first implemented a subcutaneous model. B16.F10 cells were injected into the flank of either Mcpt5-Cre^−^ R-DTA^+^ (hereafter denoted Mcpt5-Cre^−^), mast cell-sufficient mice or to Mcpt5-Cre^+^ R-DTA^+^ (hereafter denoted Mcpt5-Cre^+^) littermate controls. One or two weeks after administration of the melanoma cells, tumors were excised and assessed. As seen in Figure 1A, mast cells were abundant in tumor tissue recovered from the Mcpt5-Cre^−^ mice. Notably, mast cells were particularly abundant in the tumor stroma, at a distance (∼300-500 μm) from the tumor border. In contrast, we were not able to detect mast cells within the tumor mass. Mast cells were not found in tumor tissue taken from the Mcpt5-Cre^+^ mice, indicating that the strategy based on Mcpt5 expression efficiently targets mast cells in this setting. Moreover, this indicates that the mast cells found adjacent to the subcutaneous tumor tissue (in the Mcpt5-Cre^−^ mice) were of Mcpt5^+^ phenotype, i.e. of the CTMC subtype. The absence of mast cells did not affect the size or weight of the formed subcutaneous tumors (Figure 1B–1C), suggesting that mast cells have no major impact on the growth of melanoma tumors under these conditions.

### Lungs from Mcpt5-Cre^+^ mice retain expression of mucosa-subtype mast cell markers

**Figure 2:**
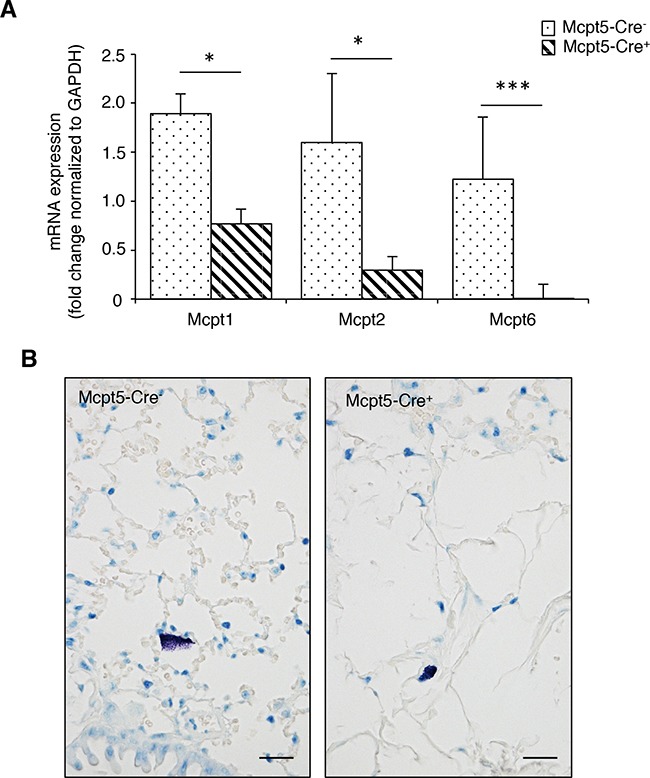
Lungs from Mcpt-5 Cre^+^ mice retain expression of mucosal type mast cell markers and contain mast cells **A.** mRNA was prepared from lungs of Mcpt5-Cre^−^ and Mcpt5-Cre^+^ mice and was analyzed by qPCR for content of transcripts for Mcpt1 (mucosal mast cell marker), Mcpt2 (mucosal mast cell marker) and Mcpt6 (marker of connective tissue type mast cells). Results are shown as mean values ± SD, n = 4, * p <0.05, ***p < 0.001. **B.** Representative images of lung sections from Mcpt5-Cre^−^ and Mcpt5-Cre^+^ mice stained with toluidine blue. Note the presence of toluidine blue-positive mast cells in lungs of both Mcpt5-Cre^−^ and Mcpt5-Cre^+^ mice. Original magnification = 400 x; Bar = 10 μm.

Our next aim was to evaluate whether mast cells contribute to the dissemination of melanoma, by focusing on a model in which melanoma cells colonize the lungs (see below). To study this, we first assessed whether the Mcpt5-Cre-based approach has consequences for the mast cell population of the lung. Previous studies have revealed that the Mcpt5-Cre approach deletes a major portion of mast cells present in skin (see also Figure 1A) and peritoneal cavity [35], i.e. at tissue locations where mast cells of the CTMC subtype dominate. However, it is not clear whether this approach deletes mast cells populating the lung. To address this question we analyzed lung tissue from Mcpt5-Cre^−^ and Mcpt5-Cre^+^ mice, respectively, for content of mRNA coding for mast cell markers. In agreement with the ablation of mast cells of the CTMC class, Mcpt6 (a CTMC marker [37]) expression was readily detected in lungs from Mcpt5-Cre^−^ mice but was undetectable in tissue from Mcpt5-Cre^+^ animals (Figure 2A). In contrast, expression of Mcpt1 and Mcpt2, encoding chymases that are regarded as markers for mast cells of the mucosal subtype [37], was detectable both in Mcpt5-Cre^−^ and Mcpt5-Cre^+^ lungs (Figure 2A). These data thus suggest that lung mast cells of the mucosal subclass are not efficiently ablated by the Mcpt5-Cre approach. In agreement with this, mast cells, most likely representing mucosal-type mast cells, were detected in lungs from Mcpt5-Cre^+^ animals (Figure 2B).

### Mast cells promote establishment of melanoma in lungs

**Figure 3:**
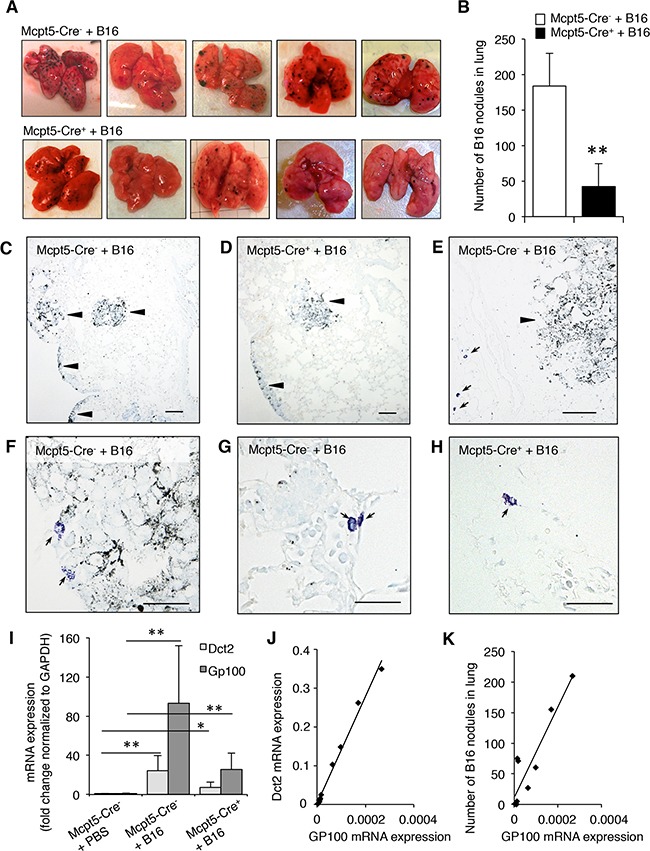
Reduced melanoma colonization in lungs from Mcpt5-Cre^+^ vs. Mcpt5-Cre^−^ mice Melanoma cells (B16.F10) were intravenously injected into Mcpt5-Cre^−^ or Mcpt5-Cre^+^ mice. After 11 days, lungs were excised. **A.** Representative images of lungs from Mcpt5-Cre^−^ and Mcpt5-Cre^+^ mice. Note the abundance of melanoma nodules in lungs from Mcpt5-Cre^−^ mice and the lower content of nodules in lungs from Mcpt5-Cre^+^ mice. **B.** Quantification of melanoma nodules; n = 8-9, ** p < 0.01. **C.** Representative image of lungs from Mcpt5-Cre^−^ animals after melanoma administration. Melanoma areas are marked by arrowheads. **D.** Representative image of lungs from Mcpt5-Cre^+^ animals after melanoma administration. Melanoma accumulations are marked by arrowheads. **E.** Image showing the presence of mast cells (arrows; toluidine blue staining) in areas at a distance from the melanoma tumors (arrowhead). (C-E): Original magnification = 100 x; Bar = 100 μm. **F.** Representative image showing that mast cells occasionally are found in the immediate vicinity to melanoma tumors. **G.** Representative image showing a higher magnification of a lung tissue section from an Mcpt5-Cre^−^ animal. Note the presence of highly granulated, toluidine blue-positive mast cells (arrows). **H.** Representative image showing a higher magnification of a lung tissue section from an Mcpt5-Cre^+^ animal. Note the presence of toluidine blue-positive mast cells (arrow). (F-H): Original magnification = 400 x; Bar = 50 μm. **I.** mRNA was extracted from lungs of melanoma-treated Mcpt5-Cre^−^ or Mcpt5-Cre^+^ mice (+B16). As a control, mRNA was extracted from naïve animals (PBS). mRNA was analyzed by qPCR for the levels of transcripts for Dct2 and Gp100; mean values ± SEM; n = 4-6; * p < 0.05, ** p < 0.01. **J.** Correlation between the expression of Gp100 and Dct2. Regression analysis: R-sq (adj) = 98.7; p < 0.001; n = 10. **K.** Correlation between expression of Gp100 and number of melanoma nodules in lungs. Regression analysis: R-sq (adj) = 82.9; p < 0.001; n =10.

To study whether mast cells influence the dissemination of melanoma, we implemented a model in which melanoma cells (B16.F10) were injected i.v. into either Mcpt5-Cre^−^ or Mcpt5-Cre^+^ littermates, followed by assessment of tumor colonization in organs. It is known that B16.F10 cells predominantly colonize lung tissue, whereas minor colonization is typically seen in other organs. In agreement with this, we noted colonization of B16.F10 cells, as nodules, in the lungs of the mice, but we were not able to detect melanoma colonization in other organs (liver, lymph nodes, spleen). As seen in Figure 3A-3C, extensive colonization with melanoma nodules was seen in lungs of Mcpt5-Cre^−^ mice. In contrast, much less colonization was seen in lungs of Mcpt5-Cre^+^ littermate animals (Figure 3A–3B, 3D). Hence, mast cells of the CTMC subtype contribute significantly to the dissemination of melanoma from blood into the lung. Similar to the subcutaneous model (see Figure 1A), mast cells were seen predominantly in areas at some distance from the tumor edge (Figure 3E) but mast cells were occasionally seen also in close contact with the tumor (Figure 3F). In Figure 3G-3H, higher magnifications of toluidine blue-stained sections are depicted. Notably, toluidine blue-positive mast cells were seen in lung tissue of both Mcpt5-Cre^−^ and Mcpt5-Cre^+^ mice.

### Expression of melanoma-specific genes in lungs of Mcpt5-Cre^−^ and Mcpt5-Cre^+^ mice

To further substantiate the impact of mast cells on melanoma colonization we assessed the expression of melanoma-specific genes in lungs from Mcpt5-Cre^−^ and Mcpt5-Cre^+^ mice. To this end we focused on Dct2 and Gp100. Dct2 encodes dopachrome tautomerase, an enzyme involved in melanin synthesis. Gp100 is a glycoprotein with a role in melanosome maturation. Quantitative real time PCR (qPCR) analysis revealed that the expression of both of these genes was minimal in naïve animals (Figure 3I). However, a profound increase in their expression was seen in Mcpt5-Cre^−^ animals after administration of melanoma cells (Figure 3I). Increased expression of Dct2 and Gp100 was also seen in Mcpt5-Cre^+^ mice, but the amplitude of induction appeared considerably lower than in Mcpt5-Cre^−^ mice (Figure 3I). Hence, these findings support that mast cells of CTMC subtype promote melanoma colonization of the lung. As seen in Figure 3J, there was a strong correlation between the expression of Dct2 and Gp100. Moreover, we observed a significant association between the number of melanoma nodules and the levels of expression of Gp100 (Figure 3K).

### Increased inflammation of the bronchoalveolar lavage fluid in Mcpt5-Cre^+^ mice

**Figure 4:**
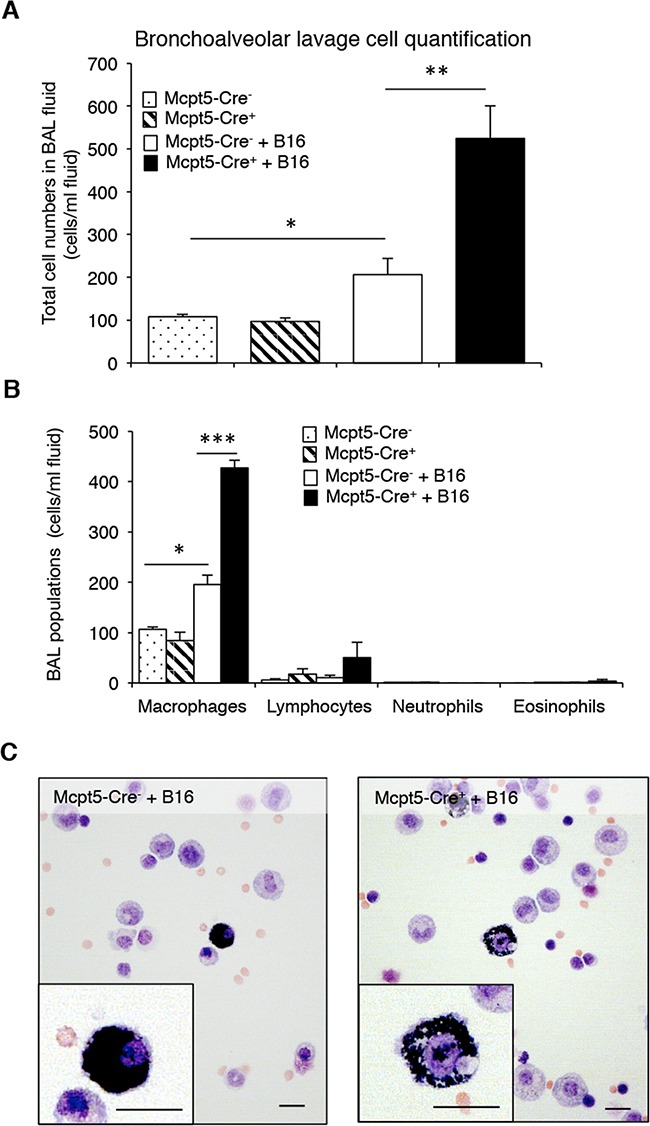
Higher inflammation in bronchoalveolar lavage fluid of melanoma-treated Mcpt5-Cre^+^ vs. Mcpt5-Cre^−^ animals Melanoma cells (B16.F10) were intravenously injected into Mcpt5-Cre^−^ or Mcpt5-Cre^+^ littermate mice. After 11 days, bronchoalveolar lavage (BAL) was performed. As controls, BAL fluids from naïve Mcpt5-Cre^−^ and Mcpt5-Cre^+^ mice were assessed. **A-B.** Total cell counts (A) and differential counting (B) of BAL fluid from either naïve or melanoma-treated Mcpt5-Cre^−^ or Mcpt5-Cre^+^ mice. Results are given as mean ± SD; n = 4; * p < 0.05, ** p < 0.01. **C.** Representative images of May-Grünwald/Giemsa-stained cytospin slides prepared from BAL fluid of melanoma-treated Mcpt5-Cre^−^ or Mcpt5-Cre^+^ mice. Note the presence of macrophages with ingested melanin (insets). Original magnification = 400 x; Bar = 10 μm.

Mast cells are strongly implicated in the regulation of inflammation, by secreting pro-inflammatory mediators but there are also indications that mast cells can suppress inflammatory responses [1, 2]. To address the possibility that mast cells affect the inflammatory response in the context of melanoma colonization, we assessed the leukocyte populations in the bronchoalveolar lavage (BAL) fluid of Mcpt5-Cre^−^ vs. Mcpt5-Cre^+^ mice, both in naïve animals and following melanoma administration. In the Mcpt5-Cre^−^ animals, the administration of melanoma cells resulted in a significant (∼2-fold) increase in the total numbers of leukocytes recovered in the BAL fluid (Figure 4A). In the Mcpt5-Cre^+^ animals, the administration of melanoma cells resulted in a much more profound increase in BAL fluid leukocytes, with over 5-fold higher total leukocyte counts in BAL fluid of melanoma-treated vs. naïve animals (Figure 4A). Differential counting showed that macrophages represented the major leukocyte population of the BAL fluid, both in naïve animals and after melanoma administration (Figure 4B–4C). Lymphocytes were also detected whereas granulocytes were essentially undetectable, both in naïve and melanoma-treated animals (Figure 4B–4C). Together, these data indicate that the administration of melanoma gives rise to a macrophage-dominated inflammatory response, which is considerably more profound in Mcpt5-Cre^+^ than in Mcpt5-Cre^−^ animals.

### Expression of genes implicated in epithelial-mesenchymal transition in Mcpt5-Cre^−^ and Mcpt5-Cre^+^ mice

**Figure 5:**
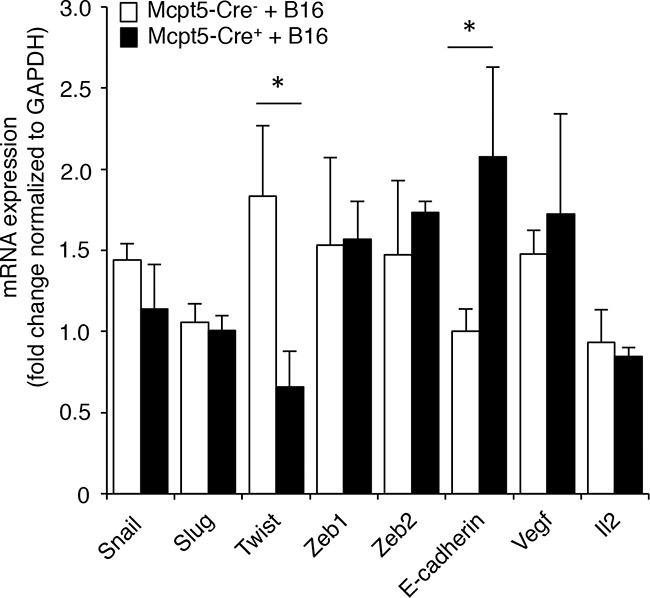
Altered expression of genes related to epithelial-mesenchymal transition in melanoma-treated Mcpt5-Cre^−^ vs. Mcpt5-Cre^+^ mice Melanoma cells (B16.F10) were intravenously injected into Mcpt5-Cre^−^ or Mcpt5-Cre^+^ mice. After 11 days, lungs were excised, followed by preparation of mRNA and qPCR analysis of levels of transcripts for Snail, Slug, Twist, Zeb1, Zeb2, E-cadherin, Vegf and Il2. Results are presented as mean ± SEM; n = 5-6, * p < 0.05.

To further address the mechanism by which mast cells influence melanoma colonization, we assessed whether the absence of Mcpt5^+^ mast cells affects the expression of genes with a potential role in melanoma establishment/dissemination. Since epithelial-mesenchymal transition (EMT) is a hallmark event occurring during tumor dissemination [38] we analyzed for the expression of genes implicated in this process. As seen in Figure 5, the expression of the EMT markers Snail, Slug, Zeb1 and Zeb2 were similar in lungs from Mcpt5-Cre^−^ vs. Mcpt5-Cre^+^ animals. The expression of Il2 and Vegf was also similar among the genotypes. In contrast, the expression of Twist, a transcription factor implicated in metastasis [39], was significantly repressed in Mcpt5-Cre^+^ animals in comparison with the fully mast cell-sufficient Mcpt5-Cre^−^ littermates. Moreover, the expression of E-cadherin, an epithelial cell marker, was markedly higher in the Mcpt5-Cre^+^ than in the mast cell-sufficient Mcpt5-Cre^−^ animals. Hence, the absence of mast cells in the Mcpt5-Cre^+^ mice causes altered expression of genes related to EMT.

## DISCUSSION

Numerous studies have addressed the role of mast cells in cancer by using animals deficient in mast cells [7-10]. However, most of these previous studies have utilized models in which mast cell-deficiency is a consequence of defective c-kit signaling. Considering that c-kit has various functions apart from regulating mast cell growth/differentiation, it is essential to ascertain that any observed phenotypes in such c-kit-defective mice are indeed due to their mast cell-deficiency as opposed to other effects related to c-kit. In light of the strong implication of c-kit (independent of mast cells) in malignancies [40], this may be a more serious issue when addressing the role of mast cells in cancer than for studies of mast cell involvement in other types of pathologies. It is therefore critical to show that the phenotype of the c-kit defective animals is reversed to that of wild type mice by mast cell reconstitution. Notably though, in studies where mouse models have been utilized for studying the role of mast cells in melanoma and other malignant processes, this critical issue has not been resolved [26-31]. Moreover, there are indications that the reconstitution approach as such can cause artifacts that confound the interpretation of the data [33, 35]. Hence, despite intense investigations using both clinical and experimental approaches, the functional impact of mast cells in cancer is still largely an open question.

In this study we addressed the role of mast cells in a model of melanoma by assessing mice where mast cells are ablated by a strategy utilizing the promoter for Mcpt5, a chymase that is highly restricted to mast cells of the CTMC subtype [36]. In agreement with the latter, previous studies have shown that the Mcpt5-Cre approach results in efficient depletion of mast cells in skin and peritoneal cavity, i.e. tissues where mast cells of the CTMCs subtype dominate over mucosal-type mast cells. Here we show that the Mcpt5-Cre strategy results in efficient ablation of mast cells in the subcutaneous compartment following melanoma administration, suggesting that mast cells of CTMC subtype dominate in this setting. Interestingly, when assessing the mast cell presence in lungs, we noted that lungs from Mcpt5-Cre^+^ animals retained substantial expression of Mcpt1 and Mcpt2, both of which are regarded as markers for mast cells of the mucosal subtype. Interestingly though, there is recent evidence showing that lung mast cells of the CTMC type, differently to CTMC-type mast cells at other tissue locations, express Mcpt1 and Mcpt2 in addition to the typical CTMC markers- Mcpt4, Mcpt5, Mcpt6, Mcpt7, Cpa3 [41]. Hence, it is likely that the partial reduction of Mcpt1 and Mcpt2 that we see in the Mcpt5-Cre^+^ animals corresponds to a reduction of CTMC-type mast cells that express Mcpt1 and Mcpt2. Most likely, the residual Mcpt1/Mcpt2 expression in the lungs of Mcpt5-Cre^+^ animals thus represents populations of mucosal-type mast cells, i.e. that the Mcpt5-Cre approach does not lead to eradication of mast cells of this subtype. This finding is thus in agreement with a previous study showing that the intestinal mucosal-type mast cell compartment is intact in Mcpt5-Cre^+^ animals [42].

Our findings show that melanoma colonization of lungs is substantially reduced in Mcpt5-Cre^+^ vs. Mcpt5-Cre^−^ animals, suggesting that mast cells of the CTMC subclass promote melanoma dissemination. Importantly, our study is the first to examine the functional impact of mast cells in melanoma by using an approach that is independent on c-kit abnormalities. By using the Mcpt5-Cre system, it has also been shown that mast cells contribute to the development of cutaneous lymphoma [43]. Hence, these findings collectively support a pro-tumorigenic role of mast cells of the CTMC type. However, the role of mucosal type mast cells in tumor settings remains to be clarified. This could potentially be achieved by targeting mucosal mast cell populations by utilizing a Cre recombinase-based system driven by mucosal mast cell marker genes, i.e. Mcpt1 or Mcpt2. However, in light of the expression of both of these genes in lung CTMCs [41], such an approach may have its limitations for deleting mucosal-type mast cell populations in all tissues.

Our findings are in agreement with several clinical studies showing an association of mast cell presence with poor prognosis in melanoma [22-24]. Hence, our findings together with such clinical documentation suggest that targeting of mast cells could represent a potential strategy for intervention with melanoma. Possible strategies for this purpose include agents that limit mast cell degranulation, thereby reducing the output of bioactive compounds stored in the mast cell granules [44]. Alternatively, individual mast cell mediators of potential impact on malignant processes, such as histamine and various proteases, may constitute potential targets [44]. As a third alternative, selective induction of mast cell apoptosis, e.g. through the use of lysosomotropic agents [45], could represent an efficient strategy to limit harmful effects of mast cells in the context of melanoma.

The mechanism by which mast cells promote melanoma colonization is intriguing. One potential scenario would be that mast cells regulate the inflammatory mechanisms that, in turn, can affect melanoma colonization. Indeed, we noted that the absence of CTMC-type mast cells resulted in a marked effect on the recruitment of inflammatory cells, as manifested by a substantial elevation of macrophages in the BAL fluid of mast cell-deficient Mcpt5-Cre^+^ mice. Potentially, this macrophage population could have an anti-tumorigenic function, hence contributing to the reduced melanoma colonization seen in the Mcpt5-Cre^+^ mice. However, in light of the notion that mast cells predominantly have pro-inflammatory functions, it was somewhat unexpected that the extent of inflammation was higher in Mcpt5-Cre^+^ mice as compared with Mcpt5-Cre^−^ littermates. At present we are not able to explain exactly how CTMC-type mast cells dampen the inflammatory response during melanoma colonization. However, a possible scenario is that mast cells down-regulate inflammation by secreting anti-inflammatory mediators that affect melanoma colonization either directly or indirectly via inhibition of other inflammatory cells. The most likely scenario is that it is the CTMCs resident in the lung tissue that account for the observed effects, but we cannot exclude an influence of CTMCs at distant sites on the progress of melanoma colonization. The latter would be in line with a recent report where it was shown that tumors could provoke inflammatory reactions at sites distant from the malignancy [46].

Interestingly, although our data support an important role for mast cells in promoting melanoma colonization of the lung, we did not see any impact of CTMC-type mast cells on the melanoma progression in the subcutaneous model. Although we cannot at present explain the differential impact of mast cells in these two settings, we can speculate that mast cells have limited capability to directly influence the growth of tumors once established (reflecting the situation in the subcutaneous model), whereas the impact of mast cells on melanoma colonization of the lung may reflect an ability of the mast cells to influence their migration into the lung tissue.

Another possible explanation for the reduced melanoma dissemination in Mcpt5-Cre^+^ animals could be that mast cells affect EMT events, based on recent findings implicating mast cells in such processes [47]. Indeed, we show that mast cell-deficiency in Mcpt5-Cre^+^ mice is associated with significant alterations in the expression of the EMT-related genes Twist and E-cadherin. Possibly, mast cell-dependent effects on the expression of these genes may thus have consequences for regulation of EMT events following melanoma administration, such that melanoma colonization is hampered in the absence of Mcpt5-expressing mast cells. Although we cannot at present explain the exact mechanism behind this finding, we may hypothesize that mast cells, when encountering melanoma cells, are activated to secrete factors that in turn can modulate the expression of EMT-related genes in adjacent cellular compartments.

The i.v. melanoma model has been frequently used in the past as a model for tumor metastasis. However, although the model reflects processes occurring in the late stages of the metastatic process it does not reflect the early phase of the metastasis, i.e. the egression of tumor cells from the primary tumor into the circulation. Taken together, our findings thus suggest a role for mast cells in the late stage of metastasis.

## MATERIALS AND METHODS

### Mice

Mast cell-deficient Mcpt5-Cre^+^ R-DTA^+^ and control littermate Mcpt5-Cre^−^ R-DTA^+^ male mice (8 - 24 weeks old) were used in all experiments [35, 36]. Genotyping was performed as described [35]. Mice were all on C57BL/6 genetic background and housed at the Swedish National Veterinary Institute, Uppsala, Sweden. All procedures for animals were approved by the Uppsala ethical committee on animal research (no C84/14).

### Cell culture

B16.F10 mouse melanoma cells were cultured in DMEM supplemented with 10% (v/v) heat-inactivated fetal bovine serum and 1 x Penicillin/Streptomycin. Cells were split 1:10 when reaching 70-90% confluency. Prior to injection, cells were trypsinized, washed with PBS and counted using Trypan blue to ensure that the cell viability was >90%.

### Melanoma models

For the subcutaneous melanoma model, 0.5 million cells resuspended in 100 μl PBS were injected subcutaneously in the hip region of mast cell-deficient Mcpt5-Cre^+^ R-DTA^+^ and control littermate Mcpt5-Cre^−^ R-DTA^+^ male mice. Mice were euthanized with isofluran and total tumor weight per animal was recorded after 14 days. Tumors from each animal were fixed in 4% paraformaldehyde/PBS. For the intravenous melanoma model, 0.5 million cells resuspended in 100 μl PBS were injected intravenously into the tail vein of mast cell-deficient Mcpt5-Cre^+^ R-DTA^+^ and control littermate Mcpt5-Cre^−^ R-DTA^+^ male mice. Mice were euthanized with isofluran and bronchoalveolar lavage (BAL) was performed by rinsing twice with 1 ml Hanks' balanced salt solution (HBSS) after 11 days. Lung tissues were excised and melanoma nodules counted. One lung lobe from each animal was then fixed in 4% paraformaldehyde/PBS and remaining lobes were snap frozen and stored at -80°C until use. Total number of BAL fluid cells was determined by haemocytometer counting.

### Histochemistry of tissues and bronchoalveolar lavage

BAL cells were collected onto cytospin slides. Samples were stained with May-Grünwald/Giemsa (Merck) as previously described [48] and subsequently rinsed in water before mounting, followed by differential counting of at least 200 cells per coded slide.

Fixed lung and subcutaneous tumor tissues were embedded in paraffin, sectioned into 6-μm sections. Samples were deparaffinized and stained with hematoxylin and eosin, toluidine blue solution (0.1% toluidine blue in 0.17 mM NaCl [pH 2]) for 2 min, and chloroacetate esterase for 15 min as previously described [48] prior to mounting. Lung and tumor tissues were evaluated by coded analyses regarding distribution of mast cells and tumor nodules from five to seven animals. All photos were taken using a NikonU brightfield microscope at original magnification 100, 200 or 400x.

### Quantitative real time PCR

Total RNA from homogenized lung tissues was isolated by using NucleoSpin RNA II (Macherey-Nagel), and first-strand cDNA was synthesized using 100 ng RNA as template by iScript cDNA synthesis kit (Bio-Rad) according to the manufacturer's instructions. Gene expression levels were determined by quantitative RT-PCR (qPCR) using the primer pairs indicated in Table 1. qPCR was performed using SYBR GreenER SuperMix (Invitrogen), 200 nM primers, and 200 ng cDNA, following the PCR cycling conditions recommended by the manufacturer. Melting curve analysis was performed at the end of every run to ensure product uniformity. The relative amount of cDNA was determined in duplicate and calculated according to the 2^−ΔΔ*C*T^ method. Glyceraldehyde 3-phosphate dehydrogenase (GAPDH) was used as housekeeping gene.

**Table 1:** Murine primers used for qPCR

Target gene	Forward sequence (5′-3′)	Reverse sequence (5′-3′)
**Gapdh**	CTC CCA CTC TTC CAC CTT CG	CCA CCA CCC TGT TGC TGT AG
**Dct2**	TCC TCC ACT CTT TTA CAG ACG	ATT CGG TTG TGA CCA ATG GG
**Gp100**	AGC ACC TGG AAC CAC ATC TA	CCA GAG GGC GTT TGT GTA GT
**Mcpt1**	GAA GGA ATG GGT CCA GAC AT	ACG GGT CAA CTT CAC ATT CA
**Mcpt2**	GCCTATCTGAAGTTCACCACTAA	TACAGTGTGCAGCAGTCATC
**Mcpt6**	CAT TGA TAA TGA CGA GCC TCT CC	CAT CTC CCG TGT AGA GGC CAG
**Snail**	CCA CTG CAA CCG TGC TTT T	CAC ATC CGA GTG GGT TTG G
**Slug**	CTC ACC TCG GGA GCA TAC AGC	TGA AGT GTC AGA GGA AGG CGG G
**Zeb1**	ACA AGA CAC CGC CGT CAT TT	GCA GGT GAG CAA CTG GGA AA
**Zeb2**	CAC CCA GCT CGA GAG GCA TA	CAC TCC GTG CAC TTG AAC TTG
**Twist**	CGG GTC ATG GCT AAC GTG	CAG CTT GCC ATC TTG GAG TC
**E-Cadherin**	CAA GGA CAG CCT TCT TTT CG	TGG ACT TCA GCG TCA CTT TG
**Vegf**	GGA GTC TGT GCT CTG GGA TT	AAC CAA CCT CCT CAA ACC GT
**Il2**	CCT GAG CAG GAT GGA GAA TTA CA	TCC AGA ACA TGC CGC AGA G

### Statistical analysis

All data were derived from at least three independent experiments and are presented as the mean ± S.D. or S.E.M., when appropriate. Statistical comparisons were performed using a two-tailed *t* test with the assumption of unequal variance. For multiple comparisons One-Way ANOVA followed by Bonferroni correction was performed. Regression analysis was performed in Minitab 17.
